# Reviewer Acknowledgments

**DOI:** 10.1002/mlf2.12103

**Published:** 2024-02-02

mLife thanks the following reviewers for their great support and contribution in 2023.

Fang Bai

Douglas H. Bartlett

Stephen L. Bearne

Jennifer Biddle

Daniela Billi

William Brazelton

Steven Burgess

Liu Cao

Fei Chen

Huaihai Chen

Xuefeng Chen

Yin Chen

Ze Chen

Fu Ci

Patrizia Contursi

Jie Cui

Violette Da Cunha

Chittaranjan Das

Xin Deng

Ye Deng

Tao Dong

Xiuzhu Dong

Abdelaziz Ed‐Dra

Hanping Feng

Clara A. Fuchsman

Nanqin Gan

Beile Gao

Haichun Gao

Jun Gong

Tingyue Gu

Xiao Han

Yiping Han

Vasili Hauryliuk

Qiang He

Qun He

Zhili He

Alfredo Herrera‐Estrella

Jie Hong

Jiong Hong

Anyi Hu

Xing Huang

Ying Huang

Dana E. Hunt

Jeongdae Im

Dieter Jahn

Aiqun Jia

Na Jia

Zhongjun Jia

Gaofei Jiang

Yongxin Jin

Yoichi Kamagata

Suhyun Kim

Jialiang Kuang

Maggie Lau Vetter

Peter R. Leavitt

Jiesi Lei

Bing Li

Bingzhi Li

Fengna Li

Fu‐Li Li

Hanyan Li

Jiangtao Li

Meng Li

Ruoyu Li

Xu Li

Yabing Li

Yanbing Li

Zhiyong Li

Jiazhang Lian

Dawei Liang

Haihua Liang

Yihan Lin

Benjamin Liu

Changhong Liu

Haiyan Liu

Huan Liu

Junyan Liu

Shuang‐Jiang Liu

Xi‐Peng Liu

Xue Liu

Yongxin Liu

Xiaozhou Lou

Ling Lu

Chunxiong Luo

Zhao‐Qing Luo

Luyan Ma

Yuxin Mao

Shinji Masuda

Prabina K. Meher

Anastasios Melis

Xiangzhou Meng

Alessio Mengoni

Sayaka Mino

Peter Neubauer

Yong Nie

Kang Ning

Ana Beatriz F. Pacheco

Sheo Shankar Pandey

Panagiotis Papatheodorou

Petra Patakova

Cheng‐Feng Qin

Jiguo Qiu

Abuduaini Rexiding

Ramon Rossello‐Mora

Muhammad Saleem

Robert Sanford

Zongze Shao

Aimee Shen

Qingtao Shen

Kaixiang Shi

Wenyu Shi

Meinhard Simon

Shihao Song

Yizhi Song

Chaomin Sun

Jun Sun

Lin Sun

Ping Sun

Yanni Sun

Clifford Swanson

Mancheng Tang

Xiao‐Feng Tang

Xuanyu Tao

Raghavan Varadarajan

S. N. Venter

Dongyu Wang

Fang Wang

Jinfeng Wang

Jun Wang

Pei Wang

Xiang‐Xi Wang

Xiaoxue Wang

Yipeng Wang

Yue Wang

Adison Wong

Thomas Wood

Ai‐Bo Wu

Chao Wu

Linwei Wu

Chuanwu Xi

Yu Xia

Xiang Xiao

Jianping Xie

Ruize Xie

Wanying Xie

Jianping Xu

Lei Xu

Liangxiong Xu

Ping Xu

Luying Xun

Aixin Yan

Chunfu Yang

Jun Yang

Sheng Yang

Yunfeng Yang

Yufeng Yao

Hongbing Yu

Fangyou Yu

Mengting Yuan

Dayi Zhang

Ju‐Yuan Zhang

Xiaohua Zhang

Yingdan Zhang

Yong Zhang

Feng Zhao

Liping Zhao

Rui Zhao

Luping Zheng

Qingfei Zheng

Xiao‐Yang Zhi

Aifen Zhou

Ning‐Yi Zhou

Peng Zhou

Zheming Zhou

Bingdong Zhu

Zhen Zhu

